# (*E*)-3-Bromo-*N*′-(2-hydr­oxy-1-naphthyl­idene)benzohydrazide

**DOI:** 10.1107/S1600536809022533

**Published:** 2009-06-17

**Authors:** Guo-Biao Cao, Xu-Hui Lu

**Affiliations:** a Department of Chemistry, Ankang University, Ankang Shanxi 725000, People’s Republic of China

## Abstract

The title compound, C_18_H_13_BrN_2_O_2_, was synthesized by the reaction of 2-hydr­oxy-1-naphthaldehyde with an equimolar quantity of 3-bromo­benzohydrazide in methanol. The dihedral angle between the benzene ring and the naphthyl ring system is 18.3 (2)°. An intra­molecular O—H⋯N hydrogen bond is observed between the phenolate O and imine N atoms. In the crystal structure, mol­ecules are linked through inter­molecular N—H⋯O and C—H⋯O hydrogen bonds, forming a chain running along [101].

## Related literature

For crystal structures of hydrazone compounds, see: Mohd Lair *et al.* (2009 ▶); Fun *et al.* (2008 ▶); Li & Ban (2009 ▶); Zhu *et al.* (2009 ▶); Yang (2007 ▶); You *et al.* (2008 ▶). For hydrazone compounds reported previously by our group, see: Qu *et al.* (2008 ▶); Yang *et al.* (2008 ▶).
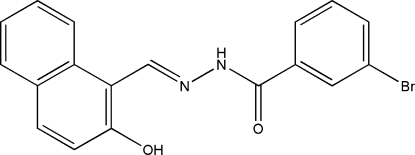


## Experimental

### 

#### Crystal data

C_18_H_13_BrN_2_O_2_
*M* *_r_* = 369.21
Monoclinic, 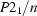

*a* = 7.257 (1) Å
*b* = 31.229 (2) Å
*c* = 7.327 (1) Å
β = 109.186 (2)°
*V* = 1568.3 (3) Å^3^
*Z* = 4
Mo *K*α radiation
μ = 2.63 mm^−1^
*T* = 298 K
0.27 × 0.24 × 0.23 mm

#### Data collection

Bruker SMART CCD area-detector diffractometer
Absorption correction: multi-scan (*SADABS*; Bruker, 2001 ▶) *T*_min_ = 0.505, *T*_max_ = 0.548
9563 measured reflections
3393 independent reflections
2177 reflections with *I* > 2σ(*I*)
*R*_int_ = 0.042

#### Refinement

*R*[*F* ^2^ > 2σ(*F* ^2^)] = 0.049
*wR*(*F* ^2^) = 0.130
*S* = 1.03
3393 reflections
212 parameters
1 restraint
H atoms treated by a mixture of independent and constrained refinement
Δρ_max_ = 0.95 e Å^−3^
Δρ_min_ = −0.74 e Å^−3^

### 

Data collection: *SMART* (Bruker, 2007 ▶); cell refinement: *SAINT* (Bruker, 2007 ▶); data reduction: *SAINT*; program(s) used to solve structure: *SHELXTL* (Sheldrick, 2008 ▶); program(s) used to refine structure: *SHELXTL*; molecular graphics: *SHELXTL*; software used to prepare material for publication: *SHELXTL*.

## Supplementary Material

Crystal structure: contains datablocks global, I. DOI: 10.1107/S1600536809022533/rz2335sup1.cif

Structure factors: contains datablocks I. DOI: 10.1107/S1600536809022533/rz2335Isup2.hkl

Additional supplementary materials:  crystallographic information; 3D view; checkCIF report

## Figures and Tables

**Table 1:** Hydrogen-bond geometry (Å, °)

*D*—H⋯*A*	*D*—H	H⋯*A*	*D*⋯*A*	*D*—H⋯*A*
O1—H1⋯N1	0.82	1.86	2.584 (4)	146
N2—H2⋯O2^i^	0.90 (3)	1.99 (2)	2.840 (4)	159 (4)
C11—H11⋯O2^i^	0.92	2.42	3.138 (4)	134

Symmetry code: (i) 
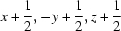
.

**Fig. 1.**
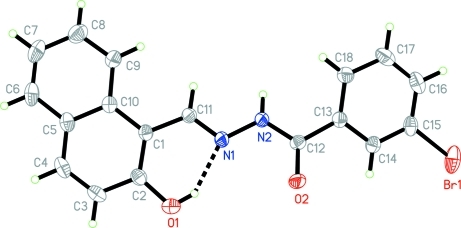
The molecular structure of the title compound with displacement ellipsoids drawn at the 30% probability level. The intermolecular O—H···N hydrogen bond is shown as a dashed line.

**Fig. 2.**
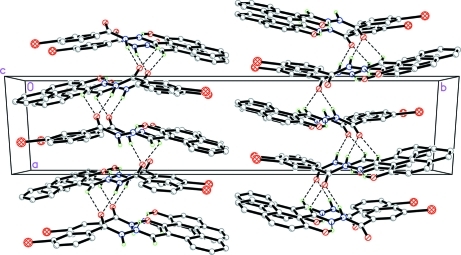
The molecular packing of the title compound, viewed along the c axis. Hydrogen bonds are drawn as dashed lines.

## supplementary crystallographic information

### Comment

Study on the crystal structures of hydrazone derivatives is a hot topic in structural chemistry. In the last few years, the crystal structures of a large number of hydrazone compounds have been reported (Mohd Lair *et al.*, 2009; Fun *et al.*, 2008; Li & Ban, 2009; Zhu *et al.*, 2009; Yang, 2007; You *et al.*, 2008). As a continuation of our work in this area (Qu *et al.*, 2008; Yang *et al.*, 2008), the title new hydrazone compound derived from the reaction of 2-hydroxynaphthaldehyde with an equimolar quantity of 3-bromobenzohydrazide is reported.

In the title compound compound (Fig. 1), the dihedral angle between the benzene ring system and the naphthyl ring is 18.3 (2)°. An intramolecular O—H···N hydrogen bond is observed between the phenolate O and imine N atoms. In the crystal structure, molecules are linked through intermolecular N—H···O and C—H···O hydrogen bonds (Table 1) to form a chain running along [101] (Fig. 2).

### Experimental

The title compound was prepared by refluxing equimolar quantities of 2-hydroxynaphthaldehyde with 3-bromobenzohydrazide in methanol. Colourless block-like crystals were formed by slow evaporation of the solution in air.

### Refinement

H2 was located in a difference Fourier map and refined isotropically, with the N–H distance restrained to 0.90 (1) Å. The other H atoms were placed in idealized positions and constrained to ride on their parent atoms, with C–H distances of 0.93 Å, O–H distance 0.82 Å, and with *U*_iso_(H) set at 1.2*U*_eq_(C) or 1.5*U*_eq_(O).

### Crystal data

**Table:**

C_18_H_13_BrN_2_O_2_	*F*(000) = 744
*M**_r_* = 369.21	*D*_x_ = 1.564 Mg m^−^^3^
Monoclinic, *P*2_1_/*n*	Mo *K*α radiation, λ = 0.71073 Å
Hall symbol: -P 2yn	Cell parameters from 2089 reflections
*a* = 7.257 (1) Å	θ = 2.5–24.1°
*b* = 31.229 (2) Å	µ = 2.63 mm^−^^1^
*c* = 7.327 (1) Å	*T* = 298 K
β = 109.186 (2)°	Block, colourless
*V* = 1568.3 (3) Å^3^	0.27 × 0.24 × 0.23 mm
*Z* = 4	

### Data collection

**Table:**

Bruker SMART CCD area-detector diffractometer	3393 independent reflections
Radiation source: fine-focus sealed tube	2177 reflections with *I* > 2σ(*I*)
graphite	*R*_int_ = 0.042
ω scans	θ_max_ = 27.0°, θ_min_ = 1.3°
Absorption correction: multi-scan (*SADABS*; Bruker, 2001)	*h* = −9→9
*T*_min_ = 0.505, *T*_max_ = 0.548	*k* = −33→39
9563 measured reflections	*l* = −9→9

### Refinement

**Table:**

Refinement on *F*^2^	Primary atom site location: structure-invariant direct methods
Least-squares matrix: full	Secondary atom site location: difference Fourier map
*R*[*F*^2^ > 2σ(*F*^2^)] = 0.049	Hydrogen site location: inferred from neighbouring sites
*wR*(*F*^2^) = 0.130	H atoms treated by a mixture of independent and constrained refinement
*S* = 1.03	*w* = 1/[σ^2^(*F*_o_^2^) + (0.0521*P*)^2^ + 1.3512*P*] where *P* = (*F*_o_^2^ + 2*F*_c_^2^)/3
3393 reflections	(Δ/σ)_max_ = 0.001
212 parameters	Δρ_max_ = 0.95 e Å^−^^3^
1 restraint	Δρ_min_ = −0.74 e Å^−^^3^

### Special details

**Table:**

Geometry. All esds (except the esd in the dihedral angle between two l.s. planes) are estimated using the full covariance matrix. The cell esds are taken into account individually in the estimation of esds in distances, angles and torsion angles; correlations between esds in cell parameters are only used when they are defined by crystal symmetry. An approximate (isotropic) treatment of cell esds is used for estimating esds involving l.s. planes.
Refinement. Refinement of F^2^ against ALL reflections. The weighted R-factor wR and goodness of fit S are based on F^2^, conventional R-factors R are based on F, with F set to zero for negative F^2^. The threshold expression of F^2^ > 2sigma(F^2^) is used only for calculating R-factors(gt) etc. and is not relevant to the choice of reflections for refinement. R-factors based on F^2^ are statistically about twice as large as those based on F, and R- factors based on ALL data will be even larger.

### Fractional atomic coordinates and isotropic or equivalent isotropic displacement parameters (Å^2^)

**Table:**

	*x*	*y*	*z*	*U*_iso_*/*U*_eq_	
Br1	0.65464 (7)	0.056963 (13)	0.64773 (9)	0.0777 (2)	
N1	0.5623 (4)	0.28551 (9)	0.3163 (4)	0.0421 (7)	
N2	0.5913 (4)	0.25667 (9)	0.4663 (4)	0.0422 (7)	
O1	0.4950 (4)	0.30515 (9)	−0.0423 (4)	0.0582 (7)	
H1	0.5018	0.2894	0.0491	0.087*	
O2	0.4065 (4)	0.20685 (8)	0.2666 (3)	0.0519 (7)	
C1	0.6106 (5)	0.35584 (11)	0.2181 (5)	0.0370 (8)	
C2	0.5493 (5)	0.34517 (12)	0.0233 (5)	0.0431 (8)	
C3	0.5414 (6)	0.37649 (14)	−0.1167 (5)	0.0548 (10)	
H3	0.5041	0.3687	−0.2463	0.066*	
C4	0.5868 (6)	0.41757 (14)	−0.0669 (6)	0.0564 (11)	
H4	0.5789	0.4377	−0.1628	0.068*	
C5	0.6461 (5)	0.43074 (12)	0.1279 (5)	0.0460 (9)	
C6	0.6938 (6)	0.47395 (13)	0.1836 (7)	0.0596 (11)	
H6	0.6865	0.4943	0.0889	0.072*	
C7	0.7495 (7)	0.48622 (14)	0.3699 (8)	0.0687 (13)	
H7	0.7765	0.5149	0.4026	0.082*	
C8	0.7662 (7)	0.45580 (14)	0.5133 (7)	0.0664 (12)	
H8	0.8070	0.4643	0.6422	0.080*	
C9	0.7234 (6)	0.41362 (12)	0.4677 (6)	0.0536 (10)	
H9	0.7361	0.3939	0.5662	0.064*	
C10	0.6602 (5)	0.39952 (11)	0.2733 (5)	0.0389 (8)	
C11	0.6256 (5)	0.32334 (11)	0.3624 (5)	0.0391 (8)	
H11	0.6829	0.3302	0.4923	0.047*	
C12	0.5114 (5)	0.21769 (11)	0.4285 (5)	0.0377 (8)	
C13	0.5620 (5)	0.18763 (11)	0.5957 (5)	0.0380 (8)	
C14	0.5772 (5)	0.14454 (11)	0.5566 (5)	0.0414 (8)	
H14	0.5539	0.1355	0.4300	0.050*	
C15	0.6263 (5)	0.11530 (12)	0.7037 (6)	0.0468 (9)	
C16	0.6536 (6)	0.12784 (14)	0.8898 (6)	0.0589 (11)	
H16	0.6840	0.1077	0.9886	0.071*	
C17	0.6359 (6)	0.17036 (15)	0.9303 (6)	0.0599 (11)	
H17	0.6546	0.1789	1.0567	0.072*	
C18	0.5903 (5)	0.20061 (12)	0.7827 (5)	0.0466 (9)	
H18	0.5790	0.2294	0.8101	0.056*	
H2	0.682 (5)	0.2631 (14)	0.580 (4)	0.080*	

### Atomic displacement parameters (Å^2^)

**Table:**

	*U*^11^	*U*^22^	*U*^33^	*U*^12^	*U*^13^	*U*^23^
Br1	0.0680 (3)	0.0401 (3)	0.1356 (5)	0.0096 (2)	0.0481 (3)	0.0197 (3)
N1	0.0472 (18)	0.0343 (15)	0.0368 (16)	0.0008 (13)	0.0029 (13)	0.0047 (13)
N2	0.0506 (19)	0.0339 (15)	0.0331 (15)	−0.0033 (13)	0.0015 (13)	0.0031 (13)
O1	0.075 (2)	0.0555 (17)	0.0404 (15)	−0.0080 (15)	0.0144 (14)	−0.0071 (13)
O2	0.0624 (18)	0.0407 (14)	0.0370 (14)	−0.0040 (12)	−0.0048 (12)	−0.0023 (11)
C1	0.0329 (18)	0.0374 (18)	0.0396 (19)	0.0012 (14)	0.0102 (14)	0.0042 (15)
C2	0.039 (2)	0.048 (2)	0.042 (2)	0.0022 (16)	0.0129 (16)	0.0051 (17)
C3	0.060 (3)	0.069 (3)	0.035 (2)	0.002 (2)	0.0138 (18)	0.0068 (19)
C4	0.057 (3)	0.061 (3)	0.055 (3)	0.011 (2)	0.023 (2)	0.025 (2)
C5	0.039 (2)	0.045 (2)	0.056 (2)	0.0066 (16)	0.0200 (17)	0.0120 (18)
C6	0.054 (3)	0.043 (2)	0.090 (3)	0.0031 (19)	0.034 (2)	0.020 (2)
C7	0.075 (3)	0.038 (2)	0.100 (4)	−0.008 (2)	0.039 (3)	−0.004 (2)
C8	0.074 (3)	0.054 (3)	0.073 (3)	−0.013 (2)	0.027 (2)	−0.012 (2)
C9	0.066 (3)	0.042 (2)	0.054 (2)	−0.0050 (19)	0.022 (2)	−0.0023 (18)
C10	0.0304 (18)	0.0401 (18)	0.047 (2)	0.0021 (14)	0.0135 (15)	0.0042 (16)
C11	0.039 (2)	0.0365 (19)	0.0364 (18)	0.0031 (15)	0.0057 (15)	0.0020 (15)
C12	0.0345 (19)	0.0372 (18)	0.0370 (19)	0.0027 (14)	0.0059 (15)	−0.0028 (15)
C13	0.0315 (18)	0.0393 (19)	0.0391 (19)	−0.0002 (15)	0.0061 (14)	0.0039 (15)
C14	0.0347 (19)	0.0401 (19)	0.048 (2)	−0.0020 (15)	0.0123 (16)	0.0012 (17)
C15	0.035 (2)	0.043 (2)	0.064 (3)	0.0002 (16)	0.0188 (18)	0.0123 (18)
C16	0.049 (3)	0.067 (3)	0.060 (3)	0.001 (2)	0.016 (2)	0.026 (2)
C17	0.057 (3)	0.082 (3)	0.038 (2)	−0.004 (2)	0.0129 (18)	0.009 (2)
C18	0.048 (2)	0.048 (2)	0.042 (2)	−0.0047 (17)	0.0140 (17)	−0.0012 (17)

### Geometric parameters (Å, °)

**Table:**

Br1—C15	1.894 (4)	C6—H6	0.9300
N1—C11	1.272 (4)	C7—C8	1.392 (6)
N1—N2	1.383 (4)	C7—H7	0.9300
N2—C12	1.338 (4)	C8—C9	1.369 (6)
N2—H2	0.90 (3)	C8—H8	0.9300
O1—C2	1.350 (4)	C9—C10	1.415 (5)
O1—H1	0.8200	C9—H9	0.9300
O2—C12	1.227 (4)	C11—H11	0.9300
C1—C2	1.389 (5)	C12—C13	1.490 (5)
C1—C10	1.435 (5)	C13—C18	1.378 (5)
C1—C11	1.444 (5)	C13—C14	1.388 (5)
C2—C3	1.405 (5)	C14—C15	1.368 (5)
C3—C4	1.345 (6)	C14—H14	0.9300
C3—H3	0.9300	C15—C16	1.369 (6)
C4—C5	1.410 (6)	C16—C17	1.375 (6)
C4—H4	0.9300	C16—H16	0.9300
C5—C6	1.419 (6)	C17—C18	1.391 (5)
C5—C10	1.423 (5)	C17—H17	0.9300
C6—C7	1.346 (6)	C18—H18	0.9300
			
C11—N1—N2	116.5 (3)	C8—C9—H9	119.4
C12—N2—N1	119.1 (3)	C10—C9—H9	119.4
C12—N2—H2	122 (3)	C9—C10—C5	117.4 (3)
N1—N2—H2	118 (3)	C9—C10—C1	123.2 (3)
C2—O1—H1	109.5	C5—C10—C1	119.4 (3)
C2—C1—C10	118.9 (3)	N1—C11—C1	121.7 (3)
C2—C1—C11	120.4 (3)	N1—C11—H11	119.2
C10—C1—C11	120.7 (3)	C1—C11—H11	119.2
O1—C2—C1	123.0 (3)	O2—C12—N2	122.7 (3)
O1—C2—C3	116.6 (3)	O2—C12—C13	121.8 (3)
C1—C2—C3	120.4 (3)	N2—C12—C13	115.5 (3)
C4—C3—C2	121.2 (4)	C18—C13—C14	119.6 (3)
C4—C3—H3	119.4	C18—C13—C12	123.2 (3)
C2—C3—H3	119.4	C14—C13—C12	117.1 (3)
C3—C4—C5	121.3 (4)	C15—C14—C13	120.2 (4)
C3—C4—H4	119.4	C15—C14—H14	119.9
C5—C4—H4	119.4	C13—C14—H14	119.9
C4—C5—C6	122.2 (4)	C14—C15—C16	120.6 (4)
C4—C5—C10	118.7 (4)	C14—C15—Br1	119.4 (3)
C6—C5—C10	119.1 (4)	C16—C15—Br1	120.0 (3)
C7—C6—C5	121.7 (4)	C15—C16—C17	119.8 (4)
C7—C6—H6	119.1	C15—C16—H16	120.1
C5—C6—H6	119.1	C17—C16—H16	120.1
C6—C7—C8	119.6 (4)	C16—C17—C18	120.2 (4)
C6—C7—H7	120.2	C16—C17—H17	119.9
C8—C7—H7	120.2	C18—C17—H17	119.9
C9—C8—C7	121.0 (4)	C13—C18—C17	119.5 (4)
C9—C8—H8	119.5	C13—C18—H18	120.2
C7—C8—H8	119.5	C17—C18—H18	120.2
C8—C9—C10	121.2 (4)		

### Hydrogen-bond geometry (Å, °)

**Table:**

*D*—H···*A*	*D*—H	H···*A*	*D*···*A*	*D*—H···*A*
O1—H1···N1	0.82	1.86	2.584 (4)	146
N2—H2···O2^i^	0.90 (3)	1.99 (2)	2.840 (4)	159 (4)
C11—H11···O2^i^	0.92	2.42	3.138 (4)	134

Symmetry codes: (i) *x*+1/2, −*y*+1/2, *z*+1/2.
